# Supplementary material for: RIG-I-like receptors direct inflammatory macrophage polarization against West Nile virus infection
Source: Nat Commun. 2019 Aug 13;10:3649. doi: 10.1038/s41467-019-11250-5 (PMC6692387; doi:10.1038/s41467-019-11250-5)
Supplement: Supplementary file 1 — Supplementary Information [file 41467_2019_11250_MOESM1_ESM.pdf]

RIG-I-like receptors direct inflammatory macrophage polarization against West Nile virus infection

Stone et al

Supplemental Figures:

Supplementary Figure 1

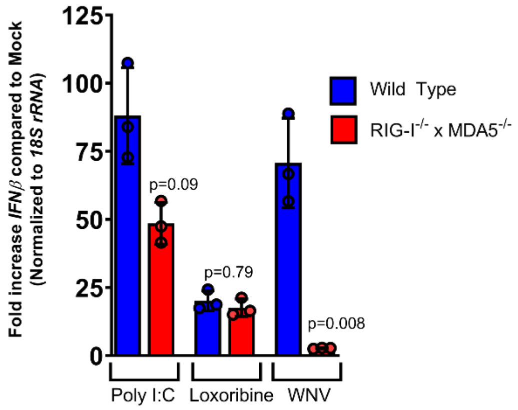

RIG-I/MDA5 DKO BMMs have intact TLR responses. WT (blue) or RIG-I/MDA5 DKO (red) BMMs were treated for 8 hours with either exogenous Poly I:C (TLR3 agonist), Loxoribine (TLR7 agonist), or infected with WNV (16 hours). RNA was then harvested and analyzed by RT-PCR for *IFNβ*. Bars are the mean fold change (as calculated by ΔΔCT) ± SEM for n=3 independent experiments. P values are shown (t-test). Source data are provided as a Source Data file.

Supplementary Figure 2

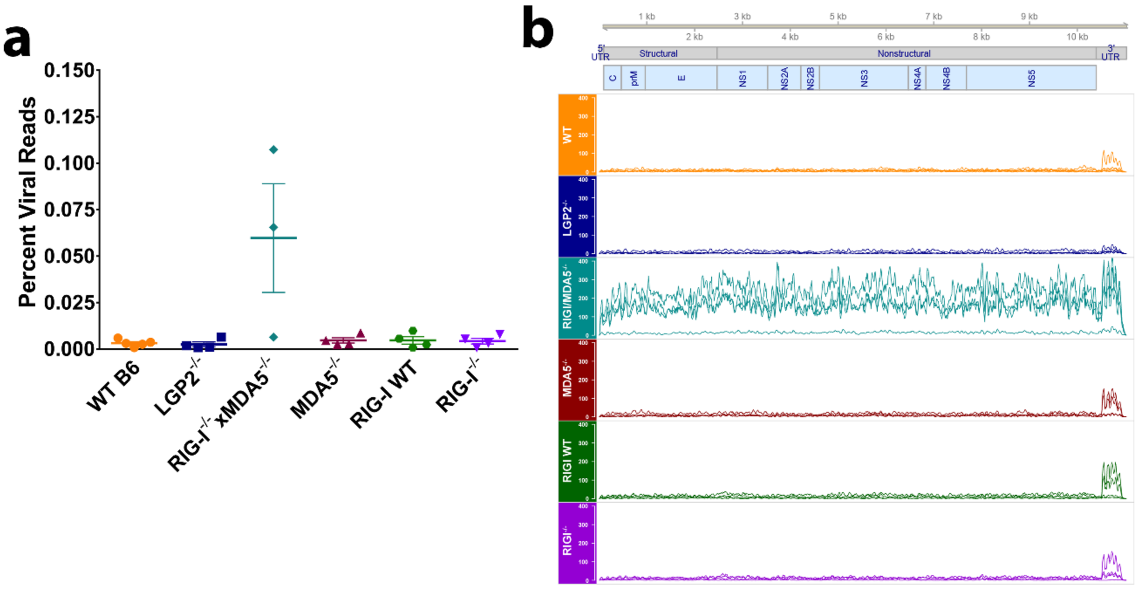

Viral Reads from RNAseq. A) Percent viral reads for each genotype. Each symbol represents a single RNAseq replicate. Lines represent the mean percent of viral reads and error bars are ± SD. B) Histogram of viral reads across the WNV genome divided by genotype. Each RNAseq replicate is shown as a unique histogram line. n=3 (DKO), 4 (LGP2$^{-/-}$, MDA5$^{-/-}$, RIG-I WT, RIG-I$^{-/-}$), or 5 (WT B6) independent infections, RNA preparations, and sequencing results. Source data are provided as a Source Data file.

Supplementary Figure 3

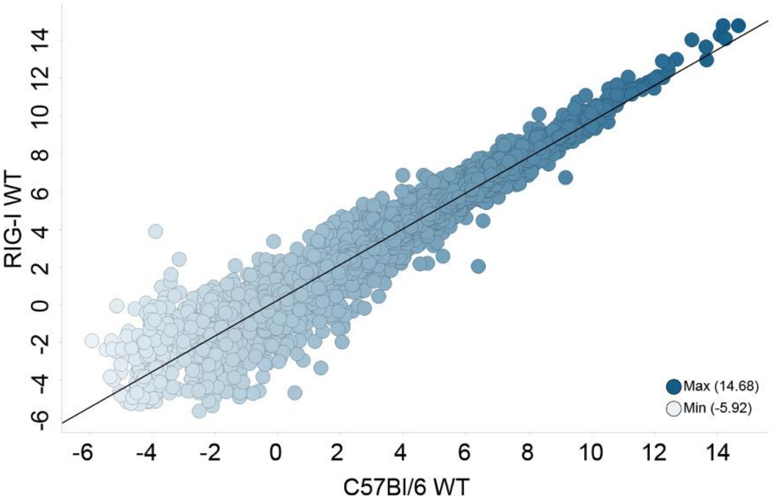

The transcriptomes of C57Bl/6 WT and RIG-I$^{+/+}$ WT are highly similar. Mock conditions for C57Bl/6 WT and RIG-I$^{+/+}$ WT were compared by logCPM (log counts per million) and plotted as a scatter plot. Line is the linear best fit line with r^2 value of 0.975. n=3 (RIG-I WT), or 4 (WT B6) independent infections, RNA preparations, and sequencing results. Source data are provided as a Source Data file.

Supplementary Figure 4

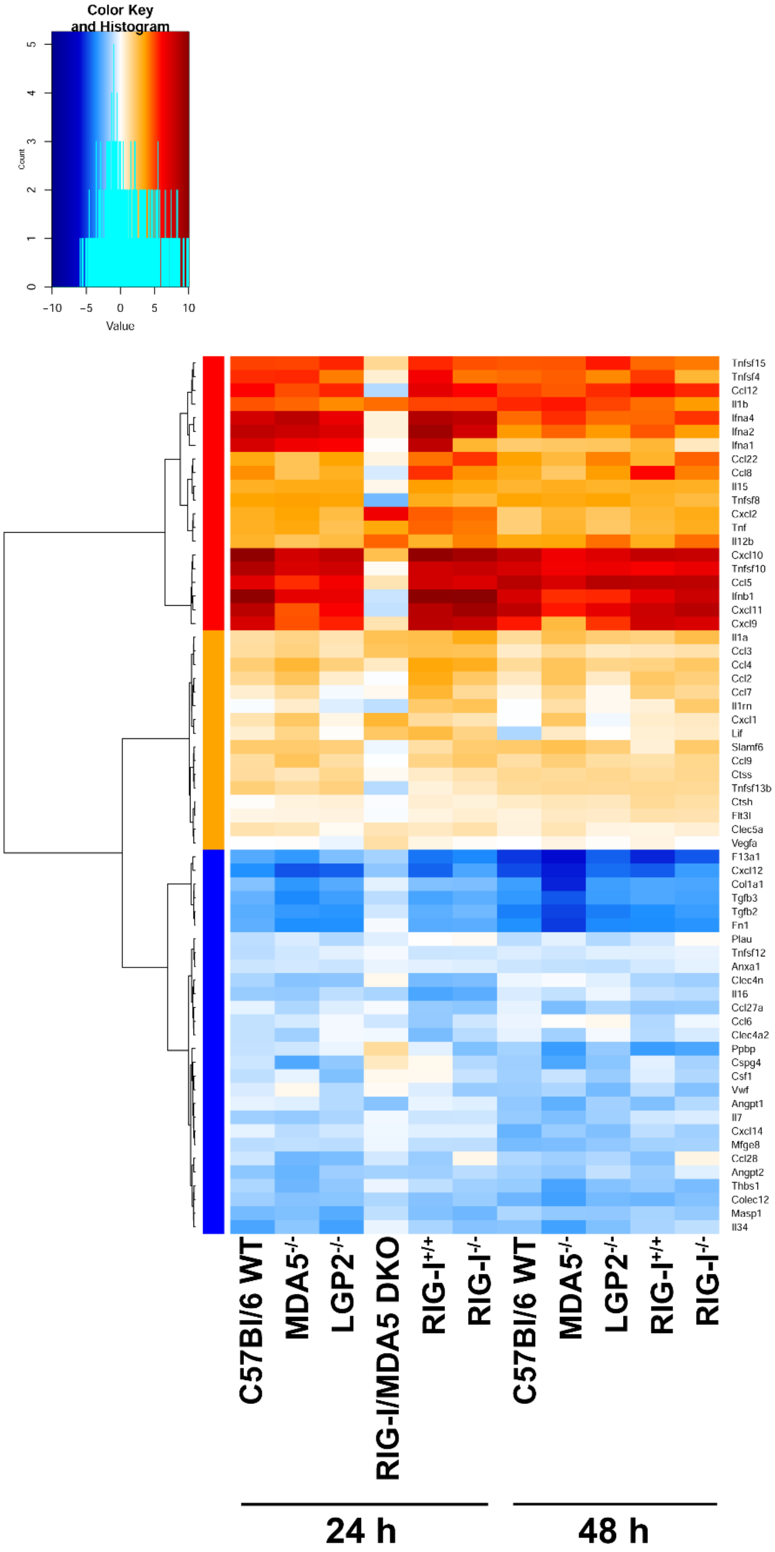
Color Key
and Histogram
Count
0
1
2
3
4
5
−10
−5
0
5
10
Value
Tnfsf15
Tnfsf4
Ccl12
Il1b
Ifna4
Ifna2
Ifna1
Ccl22
Ccl8
Il15
Tnfsf8
Cxcl2
Tnf
Il12b
Cxcl10
Tnfsf10
Ccl5
Ifnb1
Cxcl11
Cxcl9
Il1a
Ccl3
Ccl4
Ccl2
Ccl7
Il1rn
Cxcl1
Lif
Slamf6
Ccl9
Ctss
Tnfsf13b
Ctsh
Flt3l
Clec5a
Vegfa
F13a1
Cxcl12
Col1a1
Tgfb3
Tgfb2
Fn1
Plau
Tnfsf12
Anxa1
Clec4n
Il16
Ccl27a
Ccl6
Clec4a2
Ppbp
Cspg4
Csf1
Vwf
Angpt1
Il7
Cxcl14
Mfge8
Ccl28
Angpt2
Thbs1
Colec12
Masp1
Il34
C57Bl/6 WT
MDA5-/-
LGP2-/-
RIG-I/MDA5 DKO
RIG-I+/+
RIG-I-/-
C57Bl/6 WT
MDA5-/-
LGP2-/-
RIG-I+/+
RIG-I-/-
24 h
48 h

BMMs exposed to WNV alter genes involved in extracellular matrix reorganization. The differentially expressed genes from all genotypes were filtered by the Matrisome gene list. Genes that were significantly differentially expressed in at least one condition are shown on the heatmap. Heatmap is set up the same as in Figure 2C. n=3 (DKO), 4 (LGP2$^{-/-}$, MDA5$^{-/-}$, RIG-I WT, RIG-I$^{-/-}$), or 5 (WT B6) independent infections, RNA preparations, and sequencing results. Source data are provided as a Source Data file.

Supplementary Figure 5

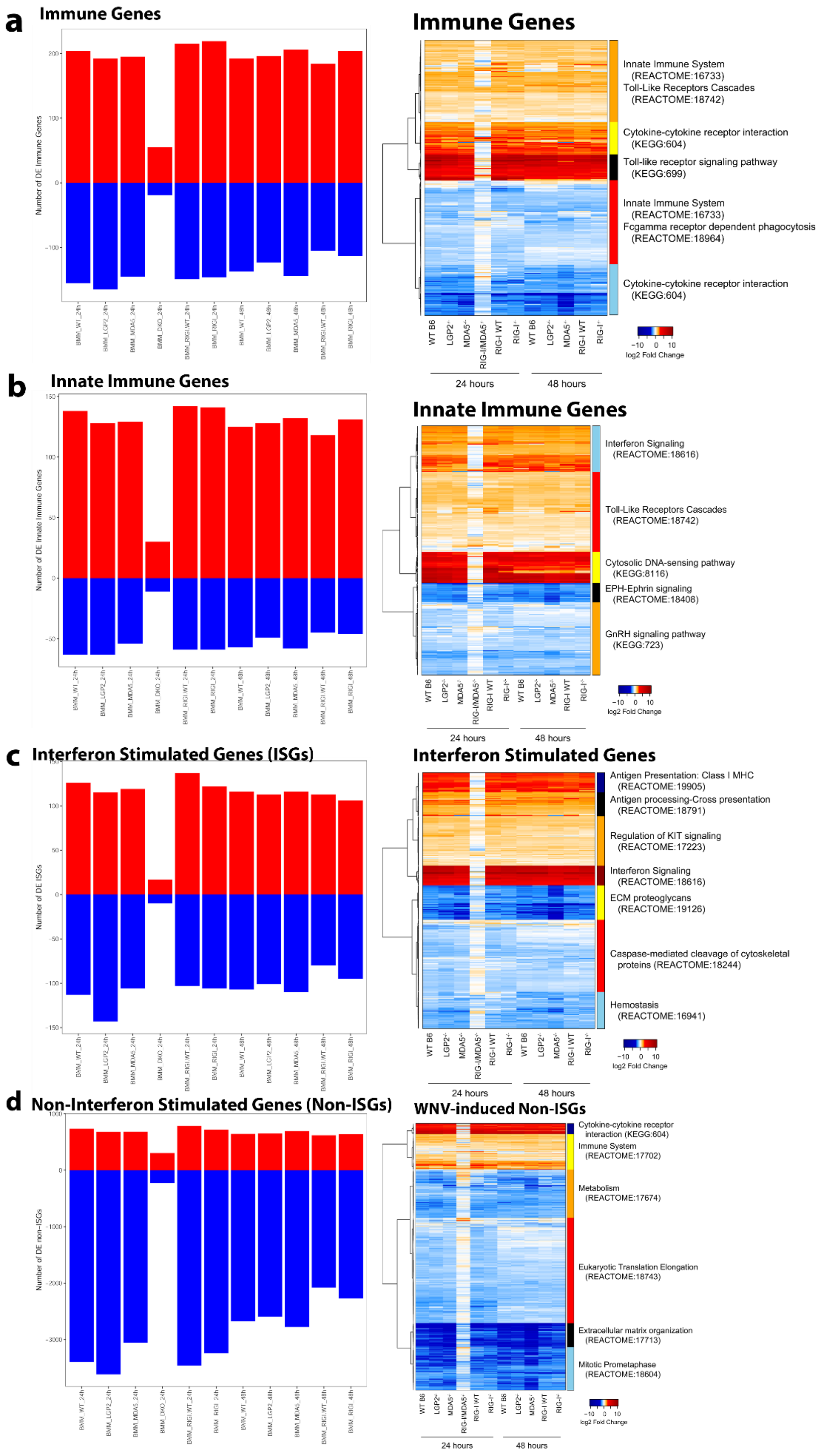
a
Immune Genes
Number of DE Immune Genes
Immune Genes
Innate Immune System
(REACTOME:16733)
Toll-Like Receptors Cascades
(REACTOME:18742)
Cytokine-cytokine receptor interaction
(KEGG:604)
Toll-like receptor signaling pathway
(KEGG:699)
Innate Immune System
(REACTOME:16733)
Fcgamma receptor dependent phagocytosis
(REACTOME:18964)
Cytokine-cytokine receptor interaction
(KEGG:604)
WT B6
LGP2-/-
MDA5-/-
RIG-I/MDA5-/-
RIG-I WT
RIG-I-/-
WT B6
LGP2-/-
MDA5-/-
RIG-I WT
RIG-I-/-
−10 0 10
log2 Fold Change
24 hours
48 hours
b
Innate Immune Genes
Number of DE Innate Immune Genes
Innate Immune Genes
Interferon Signaling
(REACTOME:18616)
Toll-Like Receptors Cascades
(REACTOME:18742)
Cytosolic DNA-sensing pathway
(KEGG:8116)
EPH-Ephrin signaling
(REACTOME:18408)
GnRH signaling pathway
(KEGG:723)
−10 0 10
log2 Fold Change
24 hours
48 hours
c
Interferon Stimulated Genes (ISGs)
Number of DE ISGs
Interferon Stimulated Genes
Antigen Presentation: Class I MHC
(REACTOME:19905)
Antigen processing-Cross presentation
(REACTOME:18791)
Regulation of KIT signaling
(REACTOME:17223)
Interferon Signaling
(REACTOME:18616)
ECM proteoglycans
(REACTOME:19126)
Caspase-mediated cleavage of cytoskeletal
proteins (REACTOME:18244)
Hemostasis
(REACTOME:16941)
−10 0 10
log2 Fold Change
24 hours
48 hours
d
Non-Interferon Stimulated Genes (Non-ISGs)
Number of DE non-ISGs
WNV-induced Non-ISGs
Cytokine-cytokine receptor
interaction (KEGG:604)
Immune System
(REACTOME:17702)
Metabolism
(REACTOME:17674)
Eukaryotic Translation Elongation
(REACTOME:18743)
Extracellular matrix organization
(REACTOME:17713)
Mitotic Prometaphase
(REACTOME:18604)
−10 0 10
log2 Fold Change
24 hours
48 hours

RLRs influence gene expression from various immune categories following WNV infection. Bar graphs (left) and heatmaps (right) representing the differentially expressed genes by genotype and time point. A single value is shown for each genotype and time point on the bar graphs. The WT DE gene list was filtered by the categories shown at the top of the graphs. A) Genes that are in the GO Term Immune Response Genes (GO:0006955), C) Innate Immune Genes (GO:0045087), D) ISGs (See Supplementary Data 4), and E) Non-Interferon Stimulated Genes (Non-ISGs) [The remaining genes on the WT gene list after removing ISGs are shown]. Heatmaps are set up the same as in Figure 2C. n=3 (DKO), 4 (LGP2$^{-/-}$, MDA5$^{-/-}$, RIG-I WT, RIG-I$^{-/-}$), or 5 (WT B6) independent infections, RNA preparations, and sequencing results.

Supplementary Figure 6

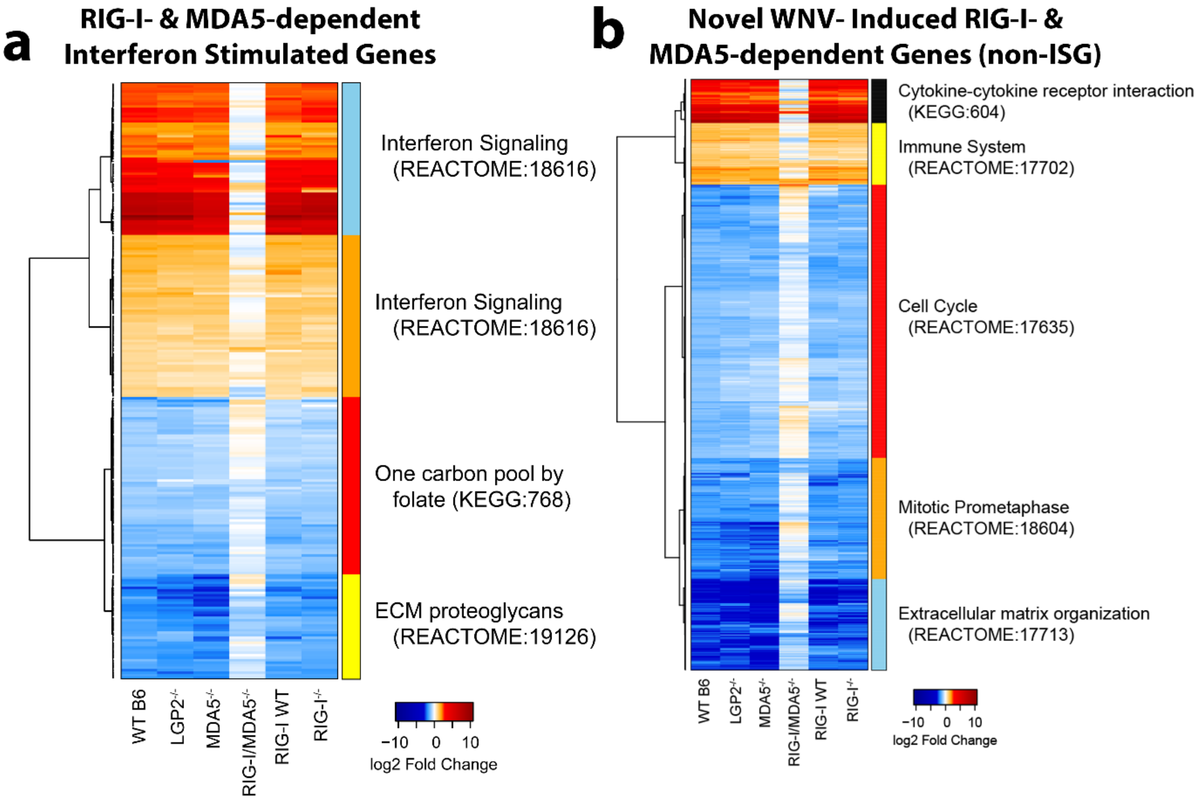

RIG-I/MDA5 DKO BMMs fail to induce changes in ISGs and Non-ISGs. The RIG-I/MDA5-dependent gene list was filtered by A) ISGs and B) Non-ISGs as described Supplementary Figure 5 and plotted by heatmap. Heatmaps are set up the same as in Figure 2C. n=3 (DKO), 4 (LGP2$^{-/-}$, MDA5$^{-/-}$, RIG-I WT, RIG-I$^{-/-}$), or 5 (WT B6) independent infections, RNA preparations, and sequencing results.

Supplementary Figure 7

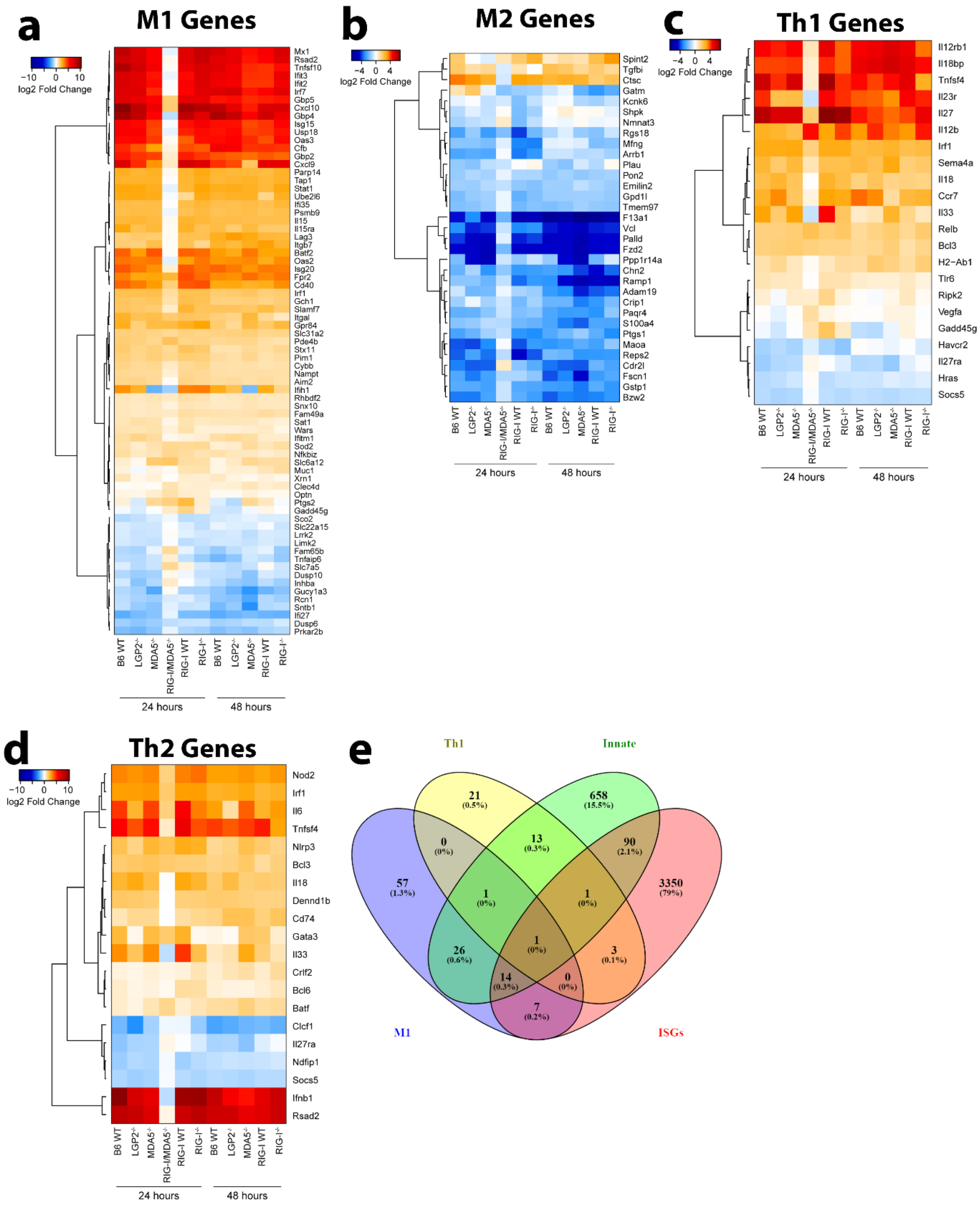
a
M1 Genes
−10 0 10
log2 Fold Change
Mx1
Rsad2
Tnfsf10
Ifit3
Ifit2
Irf7
Gbp5
Cxcl10
Gbp4
Isg15
Usp18
Oas3
Cfb
Gbp2
Cxcl9
Parp14
Tap1
Stat1
Ube2l6
Ifi35
Psmb9
Il15
Il15ra
Lag3
Itgb7
Batf2
Oas2
Isg20
Fpr2
Cd40
Irf1
Gch1
Slamf7
Itgal
Gpr84
Slc31a2
Pde4b
Stx11
Pim1
Cybb
Nampt
Aim2
Ifih1
Rhbdf2
Snx10
Fam49a
Sat1
Wars
Ifitm1
Sod2
Nfkbiz
Slc6a12
Muc1
Xrn1
Clec4d
Optn
Ptgs2
Gadd45g
Sco2
Slc22a15
Lrrk2
Limk2
Fam65b
Tnfaip6
Slc7a5
Dusp10
Inhba
Gucy1a3
Rcn1
Sntb1
Ifi27
Dusp6
Prkar2b
B6 WT
LGP2-/-
MDA5-/-
RIG-I/MDA5-/-
RIG-I WT
RIG-I-/-
B6 WT
LGP2-/-
MDA5-/-
RIG-I WT
RIG-I-/-
24 hours
48 hours
b
M2 Genes
−4 0 4
log2 Fold Change
Spint2
Tgfbi
Ctsc
Gatm
Kcnk6
Shpk
Nmnat3
Rgs18
Mfng
Arrb1
Plau
Pon2
Emilin2
Gpd1l
Tmem97
F13a1
Vcl
Palld
Fzd2
Ppp1r14a
Chn2
Ramp1
Adam19
Crip1
Paqr4
S100a4
Ptgs1
Maoa
Reps2
Cdr2l
Fscn1
Gstp1
Bzw2
B6 WT
LGP2-/-
MDA5-/-
RIG-I/MDA5-/-
RIG-I WT
RIG-I-/-
B6 WT
LGP2-/-
MDA5-/-
RIG-I WT
RIG-I-/-
24 hours
48 hours
c
Th1 Genes
−4 0 4
log2 Fold Change
Il12rb1
Il18bp
Tnfsf4
Il23r
Il27
Il12b
Irf1
Sema4a
Il18
Ccr7
Il33
Relb
Bcl3
H2−Ab1
Tlr6
Ripk2
Vegfa
Gadd45g
Havcr2
Il27ra
Hras
Socs5
B6 WT
LGP2-/-
MDA5-/-
RIG-I/MDA5-/-
RIG-I WT
RIG-I-/-
B6 WT
LGP2-/-
MDA5-/-
RIG-I WT
RIG-I-/-
24 hours
48 hours
d
Th2 Genes
−10 0 10
log2 Fold Change
Nod2
Irf1
Il6
Tnfsf4
Nlrp3
Bcl3
Il18
Dennd1b
Cd74
Gata3
Il33
Crlf2
Bcl6
Batf
Clcf1
Il27ra
Ndfip1
Socs5
Ifnb1
Rsad2
B6 WT
LGP2-/-
MDA5-/-
RIG-I/MDA5-/-
RIG-I WT
RIG-I-/-
B6 WT
LGP2-/-
MDA5-/-
RIG-I WT
RIG-I-/-
24 hours
48 hours
e
Th1
Innate
M1
ISGs
21 (0.5%)
658 (15.5%)
0 (0%)
13 (0.3%)
90 (2.1%)
57 (1.3%)
1 (0%)
1 (0%)
3350 (79%)
1 (0%)
26 (0.6%)
3 (0.1%)
14 (0.3%)
0 (0%)
7 (0.2%)

Heatmaps of the genes from the six categories shown in the radar plots. A) M1 genes, B) M2 genes, C) Th1 genes (GO:0042088 – T helper 1 type immune response), and D) Th2 genes (GO:0042092 – T helper 2 type immune response) are shown. Heatmaps are set up the same as in Figure 2C. E) Venn diagram showing the overlap amongst the Th1, M1, Innate Immune and ISG categories. Top number in each section represents the number of genes in that section and the bottom number represents the percent of the total genes in the diagram that are in that section. n=3 (DKO), 4 (LGP2$^{-/-}$, MDA5$^{-/-}$, RIG-I WT, RIG-I$^{-/-}$), or 5 (WT B6) independent infections, RNA preparations, and sequencing results. Source data are provided as a Source Data file.

Supplementary Figure 8

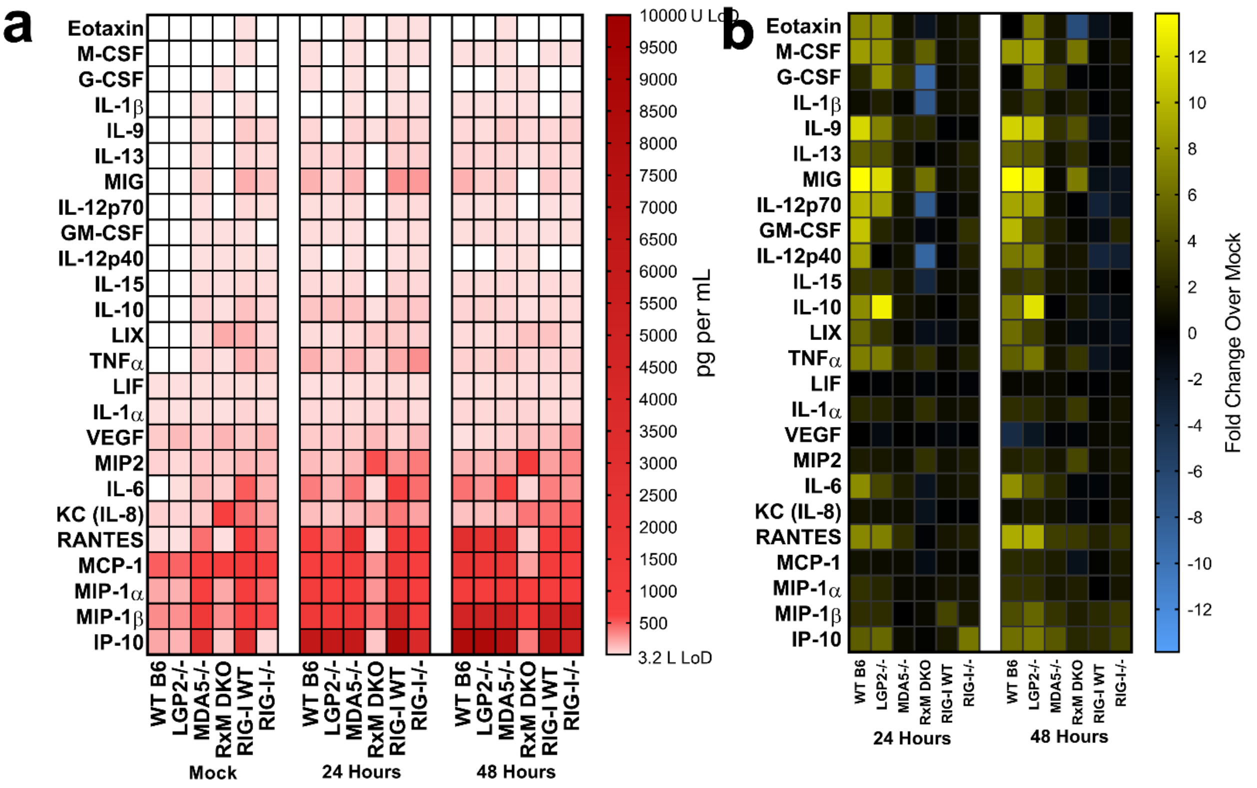

RLRs control cytokine secretion following WNV infection. A) Heatmap showing the mean pg per mL of cytokine and chemokines measured in the supernatants of BMMs infected with WNV for 24 or 48 hours, or mock infected. Each horizontal line represents a cytokine or chemokine analyzed while the columns are the genotype of each sample separated by time point. The scale is shown on right going from the lower limit of detect (L LoD) of 3.2 pg per mL to the upper limit of detection (U LoD) of 10,000 pg per mL. B) Heatmap showing the fold change over mock for each cytokine measured in the left panel. Each horizontal line represents a cytokine or chemokine analyzed while the columns are the genotype of each sample separated by time point. The scale is shown on right shows fold increase in yellow and fold decrease in blue. Black indicates no change compared to the mock sample. n=3 (DKO), 4 (LGP2$^{-/-}$, MDA5$^{-/-}$, RIG-I WT, RIG-I$^{-/-}$), or 5 (WT B6) independent experiments. Source data are provided as a Source Data file.

Supplementary Figure 9

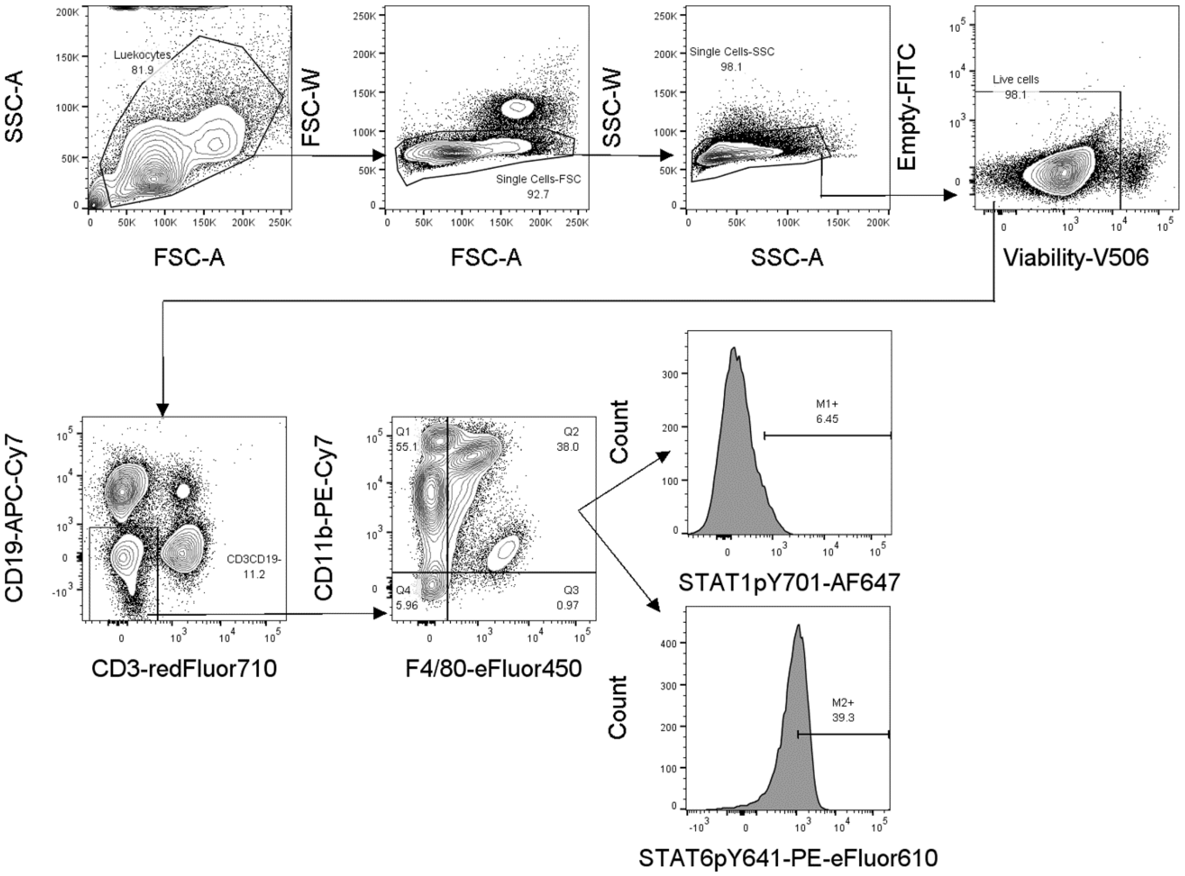

Flow cytometry gating strategy. This gating corresponds to figure 6A. Cells were gating first on size and granularity. Then singlets were select using both FSC and SSC areas versus width. Next dead cells were gated out using a live/dead viability stain where the dead cells stain positive. Next T cells (CD3) and B cells (CD19) were removed. Finally, cells that were double positive for CD11b and F4/80 were selected to analyze STAT1pY701 and STAT6pY641 expression. Representative data from n=3 independent experiments.

Supplementary Figure 10

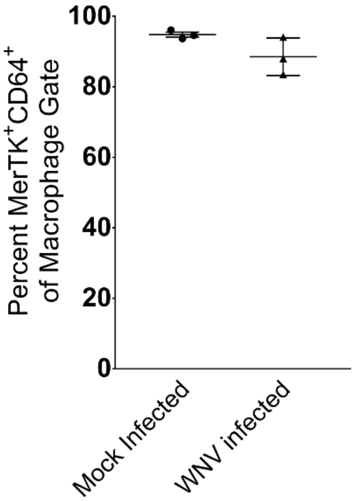

CD11b^{+}F4/80^{+} cells also express MerTK and CD64. Cells isolated from the spleen of mock or WNV infected mice were co-stained for CD11b, F4/80, MerTK, and CD64 (n=3 mice per group). B) The percent overlap between CD11b^{+}F4/80^{+} (macrophage gate) and MerTK^{+}CD64^{+} is shown with each dot representing a single mouse. Mean is shown as a solid line for each group ± SEM. n=3 mice per group. Source data are provided as a Source Data file.

Supplemental Methods

Bone marrow derived macrophages (BMMs) were generated as described in the main methods section. The cells were then lifted, counted and plated at $1x10^6$ cells per mL. Cells were allowed to adhere overnight and then infected with WNV-TX infectious clone at a multiplicity of infection (MOI) of 1 for 2 hours at which point the inoculum was removed and replaced with fresh media or treated with poly I:C (Sigma P0913) at 100ug per mL or Loxoribine (Invivogen tlrl-lox) at 1 mM. Cells were then incubated for 8 hours for the poly I:C and Loxoribine or 16 total hours for WNV infection. Cells were then harvested for RNA (Qiagen RNeasy Mini kit, 74106, as per manufacturer's instructions). RNA was submitted to RT-PCR as described in the main methods section.

WT Mock Comparisons.

To determine correlation between mock samples from different mouse backgrounds we loaded their normalized expression values (log counts per million) into a scatterplot using spotfire (version 7.11.1).

Luminex cytokine analysis.

Cellular supernatants were analyzed for the presence of cytokines using a 32-plex Milliplex kit (Millipore Sigma) as per the manufacturer's instructions.

Statistics

Supplementary Figure 1: IFNb fold change comparisons were Student's t-test adjusted for multiple comparisons between the genotypes for each stimulation DF=4: Poly I:C p=0.0913 t=3.55; Loxoribine p=0.7960 t=0.9165; WNV p=0.0081 t=7.153.
