# Supplementary material for: RIG-I-like receptors direct inflammatory macrophage polarization against West Nile virus infection
Source: Nat Commun. 2019 Aug 13;10:3649. doi: 10.1038/s41467-019-11250-5 (PMC6692387; doi:10.1038/s41467-019-11250-5)
Supplement: Supplementary file 3 — Description of Additional Supplementary Files [file 41467_2019_11250_MOESM3_ESM.pdf]

Description of Additional Supplementary Files

File name: Supplementary Data 1

Description: Differentially Regulated Genes. Genes that met the fold change minimum (2-fold) and statistical cut-off ($p>0.05$) for any condition are shown. For each gene symbol, fold change and p-value (co-expression) for each condition (genotype and time point) are shown.

File name: Supplementary Data 2

Description: Immune Genes: Genes that were found in the Total DE list (Supplementary Data 1) were then filtered by the GO term Immune Response (GO:0006955). Genes meeting this criterion for the WT DE list are included in this data. The fold change and adjusted p-value (co-expression) for each genotype are shown.

File name: Supplementary Data 3

Description: Innate Immune Genes: Genes that were found in the Total DE list (Supplementary Data 1) were then filtered by the GO term Innate Immune Response (GO:0045087). Genes meeting this criterion for the WT DE list are included in this data. The fold change and adjusted p-value (co-expression) for each genotype are shown.

File name: Supplementary Data 4

Description: Interferon-Stimulated Genes. Wild-type C57Bl/6 BMMs were stimulated with IFNβ or mock treated for 6 or 24 hours. RNA was then harvested and submitted to RNA sequencing. Comparisons of the reads in the treated condition compared to the mock-treated were used to determine fold change. Genes with a minimum of a 2-fold change (either induced or suppressed) at 24 hours were considered differentially expressed and defined as ISGs for our analysis. Gene symbols and fold change at 6 and 24 hours are shown.

File name: Supplementary Data 5

Description: Interferon-Stimulated DE Genes: Genes that were found in the Total DE list (Supplementary Data 1) were then filtered by the gene list of Supplementary Data 4 (ISGs). Genes meeting this criterion for the WT DE list are included in this data. The fold change and adjusted p-value (co-expression) for each genotype are shown.

File name: Supplementary Data 6

Description: Non- Interferon Stimulated Genes: Genes from the Total DE list (Supplementary Data 1) were filtered by the gene list of Supplementary Data 4 (ISGs) where the genes matching the list from Supplementary Data 4 were removed. The remaining genes from the WT DE list are included in this data. The fold change and adjusted p-value (co-expression) for each genotype are shown.

File name: Supplementary Data 7

Description: RLR-dependent Genes. To determine the RLR-dependent genes, we removed the genes that were differentially expressed in each RLR-Knockout from the WT DE genes. The remaining genes are genes that depend upon each RLR for their differential expression in the context of WNV infection. Genes that met this criterion for each genotype are shown. The lists are separated by time point, and by induced (up) or suppressed genes (down).

File name: Supplementary Data 8

Description: M1 DE Genes: Genes that were found in the Total DE list (Supplementary Data 1) were then filtered by the M1 defining gene list as described in Becker et al. [22]. Genes meeting this criterion for the WT DE list are included in this data. The fold change and adjusted p-value (co-expression) for each genotype are shown.

File name: Supplementary Data 9

Description: M2 DE Genes: Genes that were found in the Total DE list (Supplementary Data 1) were then filtered by the M2 defining gene list as described in Becker et al. [22]. Genes meeting this criterion for the WT DE list are included in this data. The fold change and adjusted p-value (co-expression) for each genotype are shown.

File name: Supplementary Data 10

Description: T Helper 1 DE Genes: Genes that were found in the Total DE list (Supplementary Data 1) were then filtered by the GO term T Helper 1 Type Immune Response (GO:0042088). Genes meeting this criterion for the WT DE list are included in this data. The fold change and adjusted p-value (co-expression) for each genotype are shown.

File name: Supplementary Data 11

Description: T Helper 2 DE Genes: Genes that were found in the Total DE list (Supplementary Data 1) were then filtered by the GO term T Helper 2 Type Immune Response (GO: 0042092). Genes meeting this criterion for the WT DE list are included in this data. The fold change and adjusted p-value (co-expression) for each genotype are shown.
