# Supplementary material for: RIG-I-like receptors direct inflammatory macrophage polarization against West Nile virus infection
Source: Nat Commun. 2019 Aug 13;10:3649. doi: 10.1038/s41467-019-11250-5 (PMC6692387; doi:10.1038/s41467-019-11250-5)
Supplement: Supplementary file 15 — Reporting Summary [file 41467_2019_11250_MOESM15_ESM.pdf]

naturerescarch

Corresponding author(s): Michael J Gale, Jr.; Amy EL Stone

Reporting Summary

Nature Research wishes to improve the reproducibility of the work that we publish. This form provides structure for consistency and transparency in reporting. For further information on Nature Research policies, see Authors & Referees and the Editorial Policy Checklist.

Statistical parameters

When statistical analyses are reported, confirm that the following items are present in the relevant location (e.g. figure legend, table legend, main text, or Methods section).

n/a	Confirmed	
☐	☒	The exact sample size (n) for each experimental group/condition, given as a discrete number and unit of measurement
☐	☒	An indication of whether measurements were taken from distinct samples or whether the same sample was measured repeatedly
☐	☒	The statistical test(s) used AND whether they are one- or two-sided<br>*Only common tests should be described solely by name; describe more complex techniques in the Methods section.*
☐	☒	A description of all covariates tested
☐	☒	A description of any assumptions or corrections, such as tests of normality and adjustment for multiple comparisons
☐	☒	A full description of the statistics including central tendency (e.g. means) or other basic estimates (e.g. regression coefficient) AND variation (e.g. standard deviation) or associated estimates of uncertainty (e.g. confidence intervals)
☐	☒	For null hypothesis testing, the test statistic (e.g. F, t, r) with confidence intervals, effect sizes, degrees of freedom and P value noted<br>*Give P values as exact values whenever suitable.*
☒	☐	For Bayesian analysis, information on the choice of priors and Markov chain Monte Carlo settings
☒	☐	For hierarchical and complex designs, identification of the appropriate level for tests and full reporting of outcomes
☒	☐	Estimates of effect sizes (e.g. Cohen's d, Pearson's r), indicating how they were calculated
☐	☒	Clearly defined error bars<br>*State explicitly what error bars represent (e.g. SD, SE, CI)*

Our web collection on statistics for biologists may be useful.

Software and code

Policy information about availability of computer code

Data collection	Data was provided by Expression Analysis.
Data analysis	All software tools (R/Bioconductor, star, cut adapt, ht-seq) are free and open source (software links are below). Sequencing data is in Gene Expression Omnibus (GEO) under accession GSE104817. R markdown data reports were generated for data analysis and figure reproducibility. They can be found here:<br>http://stone.galelab.org/<br><br>Links to software:<br>STAR aligner:<br>https://github.com/alexdobin/STAR<br>Bowtie2:<br>http://bowtie-bio.sourceforge.net/bowtie2/index.shtml<br>HTseq:<br>https://github.com/simon-anders/htseq<br>FASTQC:<br>https://www.bioinformatics.babraham.ac.uk/projects/fastqc/<br>Cutadapt:<br>https://github.com/marcelm/cutadapt

Venny
http://bioinfogp.cnb.csic.es/tools/venny/index.html

For manuscripts utilizing custom algorithms or software that are central to the research but not yet described in published literature, software must be made available to editors/reviewers upon request. We strongly encourage code deposition in a community repository (e.g. GitHub). See the Nature Research guidelines for submitting code & software for further information.

Data

Policy information about availability of data

All manuscripts must include a data availability statement. This statement should provide the following information, where applicable:

- Accession codes, unique identifiers, or web links for publicly available datasets
- A list of figures that have associated raw data
- A description of any restrictions on data availability

Sequencing data is in Gene Expression Omnibus (GEO) under accession GSE104817.

Field-specific reporting

Please select the best fit for your research. If you are not sure, read the appropriate sections before making your selection.

☒ Life sciences ☐ Behavioural & social sciences ☐ Ecological, evolutionary & environmental sciences

For a reference copy of the document with all sections, see nature.com/authors/policies/ReportingSummary-flat.pdf

Life sciences study design

All studies must disclose on these points even when the disclosure is negative.

Sample size	Sample size for mouse experiments were determined using a standard power calculation based on estimated effect size. Sample sizes were approved by IACUC and used through out the entire manuscript.
Data exclusions	The RIG-IxMDA5 DKO 48hour RNAsequencing was excluded due to the samples failing to pass quality control. No other samples were excluded.
Replication	Experiments were performed in replicate and data was combined for presentation. n numbers are included to indicate the number of replicates per panel.
Randomization	Littermate mice from each genotype were randomized into control (mock infection) and experimental (WNV infection) groups based on random caging at weaning.
Blinding	Blinding was used for RNAseq analysis using non-biased data analysis methods. Animal studies were not blinding as per IACUC recommendations for monitoring.

Reporting for specific materials, systems and methods

Materials & experimental systems

n/a	Involved in the study	
☐	☒	Unique biological materials
☐	☒	Antibodies
☒	☐	Eukaryotic cell lines
☒	☐	Palaeontology
☐	☒	Animals and other organisms
☒	☐	Human research participants

Methods

n/a	Involved in the study	
☒	☐	ChIP-seq
☐	☒	Flow cytometry
☒	☐	MRI-based neuroimaging

Unique biological materials

Policy information about availability of materials

Obtaining unique materials	All unique materials are available from generating investigators.

Antibodies

Antibodies used

Goat polyclonal anti-West Nile Virus NS3 R&D Systems Cat#BAF2907; RRID: AB_2215927
Rabbit Monoclonal anti-RIG-I (D14G6) Cell Signaling Technologies Cat#3743S; RRID: AB_2269233
Rabbit polyclonal anti-MDA5 ProSci Cat#4037; RRID: AB_735447
Rabbit polyclonal anti-LGP2 Proteintech Group Cat#11355-1-AP; RRID: AB_2092319
Goat polyclonal anti-Actin (I-19) Santa Cruz Biotechnology, Inc Cat#sc-1616; RRID: AB_630836
Rat anti-CD283 (TLR3) PE Biolegend Cat#141903; lot#B183765; RRID: AB_10895749
Mouse anti-CD287 (TLR7) PE (clone: A94B10) BD Biosciences Cat#565557 lot#5117683
Rat anti-CD11b PE-Cy7 (clone: M1/70) Thermo Fisher Scientific Cat#25-01112-82; lot#E07514-1633; RRID: AB_469588
Rat anti-MHCII AlexaFluor700 (clone: M5/114.15.2) Thermo Fisher Scientific Cat#56-5321-82; lot#E09021-1631; RRID:AB_494009
Humanized E16 mouse anti-WNV E Michael Diamond's Lab
Rat anti-CD19 APC-Cy7 (clone: 6D5) Biolegend Cat#115530; lot#B228154;RRID: AB_830707
Rat anti-CD3 redFluor710 (Clone: 17A2) Tonbo Biosciences Cat#80-0032-U100; lot# C003202516803; RRID: AB_2621971
Rat anti-F4/80 eFluor450 (clone: BM8) Thermo Fisher Scientific Cat#48-4801-80 lot#4278803; RRID: AB_1548756
Mouse Anti-phospho-STAT1 (pY701) (clone: 4a) BD Biosciences Cat#612597; lot# 7080509; RRID: AB_399880
Mouse anti-phosoho-STAT6 (pY641) (Clone: CHI2S4N) Thermo Fisher Scientific Cat#61-9013-41; lot# 4329183; RRID: AB_2574673
Arm. Hamster anti-CD11c PerCP-Cy5.5 (N418) Thermo Fisher Scientific Cat # 45-0114-80; Lot#4299461
Rat anti-MerTK SuperBright 702 (DS5MMER) Thermo Fisher Scientific Cat#67-5751-82; lot#1942740
Rat anti-MerTK SuperBright 600 (DS5MMER) Thermo Fisher Scientific Cat#63-5751-82; lot#4345210
Rat anti-CD206 BV605 (C068C2) Biolegend Cat#141721; lot#B248321
Mouse anti-CD64 BV786 (X54-5/7.1) BD Biosciences Cat#741024; lot#8053613
Rat anti-CD14 PE (Sa2-8) Thermo Fisher Scientific Cat#12-0141-82; lot#4344716
Rat anti-CD45 SuperBright 645 (30-F11) Thermo Fisher Scientific Cat#64-0451-82; lot#4339223

Validation

Antibodies from commercial vendors were tested on known positive samples. Antibody from our collaborators were validated in previous publications.

Animals and other organisms

Policy information about studies involving animals; ARRIVE guidelines recommended for reporting animal research

Laboratory animals

The mice used in these studies were healthy sex-matched females and males between 8-10 weeks in age that were naïve to any previous experimentation. The C57Bl/6J (WT) mice were purchased from Jackson Laboratories. All other genotypes used were bred in-house. MDA5 /-, and LGP2-/- have a 100% C57Bl/6J background, while the RIG-I-/-, RIG-I+/+ WT, and DKO all have a mixed C57Bl/6J and 129x1/SvJ background (F3 backcross from 129x1/SvJ to C57Bl/6). At the time of the experiments, mice weighed between 16.00 – 26.00g.

Wild animals

The study did not involve wild animals.

Field-collected samples

The study did not involve field-collected samples.

Flow Cytometry

Plots

Confirm that:

☒ The axis labels state the marker and fluorochrome used (e.g. CD4-FITC).

☒ The axis scales are clearly visible. Include numbers along axes only for bottom left plot of group (a 'group' is an analysis of identical markers).

☒ All plots are contour plots with outliers or pseudocolor plots.

☒ A numerical value for number of cells or percentage (with statistics) is provided.

Methodology

Sample preparation

Spleens, brains, and IP lavage were collected from mice day 6 post-infection for analysis. Half of each organ (spleen and brain) was lysed using the Percellys lysis system and used for plaque forming unit (PFU) assay. Half of the spleens were processed for flow cytometer by disruption using the GentleMacs system (Miltenyi), then cells were stained as described below. Half of the brains were mashed through a 0.7um tissue sieve (Bellco) and submitted to a hypertonic percoll gradient (1:10 10XPBS to Percoll [GE]) to remove excess myelin. Cells were then stained for flow cytometry.

Instrument

Cells were acquired on a five laser LSRII flow cytometer (BD Biosciences)

Software

Collection software was BD FACSDIVA. Flow cytometry data were analyzed using the FlowJo Software (TreeStar).

Cell population abundance	Sorting was not used in this study
Gating strategy	Gating strategy involved a large FSC/SSC gate followed by exclusion of doublets by FSC-A x FSC-W and SSC-A x SSC-W. Dead cells were then excluded based on staining with a fixable live/dead stain. Other gating is presented in the figure set with gates determined by isotype control samples and contour density population identification.

☒ Tick this box to confirm that a figure exemplifying the gating strategy is provided in the Supplementary Information.
